# Floseal-induced small bowel obstruction

**DOI:** 10.1093/jscr/rjac444

**Published:** 2022-09-28

**Authors:** Hamza Ashraf, Nicholas Low, Calista Spiro, Benjamin Keong

**Affiliations:** Department of General Surgery, Austin Health, Melbourne, Victoria, Australia; Department of General Surgery, Austin Health, Melbourne, Victoria, Australia; Department of General Surgery, Austin Health, Melbourne, Victoria, Australia; Department of General Surgery, Austin Health, Melbourne, Victoria, Australia

**Keywords:** small bowel obstruction, Floseal, haemostatic agent, laparoscopic surgery

## Abstract

Floseal is a haemostatic agent designed to augment the body’s natural clotting cascade response. We describe the first case in over a decade, and the first case with intra-operative images of early post-operative small bowel obstruction (SBO) associated with Floseal use in general surgery. A previously well man in his 30s underwent laparoscopic appendicectomy for clinical acute appendicitis. Floseal was applied to the right lateral abdominal wall for haemostasis. He developed a mechanical SBO, with diagnostic laparoscopy confirming a transition point between the caecum and terminal ileum, adherent to the area of the previously applied Floseal. He underwent adhesiolysis and uneventful recovery. We propose Floseal may exacerbate early post-operative inflammation and provide a nidus for early adhesion formation. We recommend removing excess Floseal not incorporated in the haemostatic clot and to consider Floseal as a differential in early post-operative SBO.

## INTRODUCTION

Adjuvant haemostatic agents have found increasing use in surgery, particularly in minimally invasive and laparoscopic surgery [1, 2]. Different haemostatic agents have entered the market with varying mechanisms of action, with the common pathway being to increase haemostasis by augmenting the body’s natural clotting cascade response. Floseal [3] is a haemostatic agent which combines a human-derived thrombin component with a bovine-derived gelatin matrix and is applied directly to the site of bleeding. Reported adverse effects of Floseal use include infection, rash, confusion, hypotension and atrial fibrillation [3].

Small bowel obstruction (SBO) is a relatively common post-operative complication in general surgery. Although conservative management is often successful, 10–30% of patients require re-operation, with adhesions the most common aetiology of post-operative SBO [4]. Common pathophysiological events contributing to early adhesive SBO include the body’s inflammatory healing response as well as adhesiogenesis [5].

We describe the first case in over a decade, and the first case with intra-operative images, of post-operative SBO associated with Floseal use in general surgery.

## CASE REPORT

A previously healthy man in his 30s underwent laparoscopic appendicectomy for clinical acute appendicitis. Intra-operative findings included an acutely inflamed appendix with multi-focal areas of necrosis, albeit with a healthy base. Laparoscopic appendicectomy was performed without intra-operative complication. One vial of Floseal was applied to the right lateral abdominal wall to achieve haemostasis, covering the raw, oozy surface of the previously located appendix.

**Figure 1:**
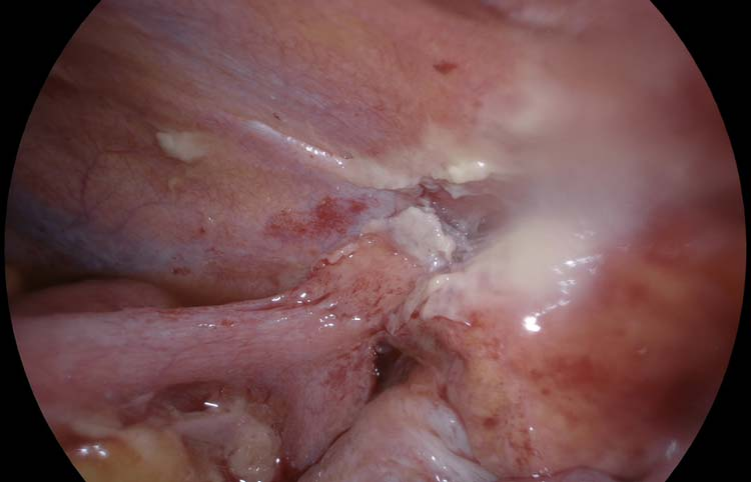
Small bowel obstruction transition point between terminal ileum, caecum and area of previously applied Floseal on abdominal wall.

Post-operatively, the patient reported two days of minimal flatus, bowels not opening, increasing nausea and distended abdomen, which did not respond to 48 hours of conservative management with nil by mouth, aperients, nasogastric tube for decompression and gastrografin follow-through. Post-operative SBO was diagnosed with computer tomography (CT) abdomen on post-operative day 4. Diagnostic laparoscopy on post-operative day 5 demonstrated distended loops of small bowel with a clear transition point between the caecum and terminal ileum, a part of which was adherent to the area of the previously applied Floseal on the right lateral abdominal wall (see Fig. 1). The bloodless fold of Treves was folded anterior to the ileocaecal valve forming a point of obstruction, with no other adhesions or points of obstruction. Adhesiolysis between the caecum, terminal ileum and abdominal wall was performed, with the patient making an otherwise uneventful recovery and discharge from hospital.

## DISCUSSION

Three previous case reports, between 2009 and 2011, described SBO after Floseal use in laparoscopic surgery. Hobday *et al*. described SBO requiring bowel resection on post-operative day 6 after Floseal was used during laparoscopic gynaecological surgery [4]. Suzuki *et al.* reported two cases of laparoscopic gynaecological surgery, with SBO developing on post-operative day 3 without requiring resection [6]. Finally, Clapp described three patients with SBO developing on post-operative day 8, with one case after bariatric surgery [5]. The majority of literature in this area is sourced from gynaecological surgery, whereas our case was the second to describe SBO following general surgery where published literature is minimal. Our case is also the first to include intra-operative images of the transition point at the area of applied Floseal.

Tawfic previously described an intense eosinophil-rich inflammatory reaction after Floseal use in gynaecological surgery contributing to post-operative pelvic pain [7]. We propose that the gelatin matrix and thrombin component of Floseal may exacerbate the inflammatory reaction involved in early post-operative healing, possibly through an eosinophil-mediated reaction as described by Tawfic, as well as providing a nidus for early adhesion formation. The manufacturer encourages removing excess Floseal not incorporated in the haemostatic clot by gentle irrigation [3], which was not performed in our case or described in previous case reports.

The paucity of published reports of SBO after Floseal use in general surgery should not discount its likelihood as a cause. Our case report serves as a reminder to not only ensure correct and judicious use of adjuvant haemostatic agents but to also consider the use of Floseal and other adjuvant haemostatic agents as differentials in early post-operative SBO following general surgery.
